# Social Participation and Elder Mistreatment in a National Sample of Older Adults

**DOI:** 10.1093/geroni/igab046.582

**Published:** 2021-12-17

**Authors:** Emmy Yang, Nadra Lisha, Ashwin Kotwal, Jaclyn Wong, Alison Huang

**Affiliations:** 1 Icahn School of Medicine at Mount Sinai, New York, New York, United States; 2 University of California San Francisco, San Francisco, California, United States; 3 University of California San Francisco, University of California San Francisco, California, United States; 4 University of South Carolina, Columbia, South Carolina, United States

## Abstract

Little is known about how social participation influences older adults' susceptibility to elder mistreatment. We conducted a cross-sectional analysis of a national probability sample of community-dwelling U.S. adults from 2015-2016 (1,268 women and 973 men; mean age 75 and 76 years, respectively; 82% non-Hispanic white). Frequency of participation in formal activities (community meetings, religious services, and volunteering) and informal social activities (socializing with friends and family) was assessed by questionnaire. Additional measures assessed emotional, physical, and financial mistreatment since age 60. Multivariable logistic regression examined associations between social participation and elder mistreatment, adjusting for age, race/ethnicity, education, and comorbidity. Forty percent of women and 22% of men reported at least one form of mistreatment (emotional, physical, or financial). Women reporting at least monthly formal social participation were more likely to report emotional mistreatment (adjusted odds ratio (AOR) 1.57, 95% confidence interval (CI) 1.08-2.29) and financial mistreatment (AOR 1.56, 95% CI 1.02-2.38) than women with less frequent engagement. Older women who socialized at least weekly were more likely to report emotional mistreatment (AOR 0.59, 95% CI 0.44-0.78) and financial mistreatment (AOR 0.59, 95% CI 0.42-0.85). These associations were not seen among older men. Frequent social engagement in the community does not preclude risk for elder mistreatment, and informal socializing may be associated with decreased exposure to certain forms of mistreatment. Assessment of older adults’ social activities may help guide strategies for detecting and mitigating elder mistreatment in the community.

